# Exploring the beneficial effects of GHK-Cu on an experimental model of colitis and the underlying mechanisms

**DOI:** 10.3389/fphar.2025.1551843

**Published:** 2025-07-02

**Authors:** Shuzhen Mao, Jiahui Huang, Junyan Li, Fang Sun, Qilian Zhang, Qing Cheng, Wei Zeng, Dongya Lei, Shiyan Wang, Jing Yao

**Affiliations:** ^1^ Jining Key Laboratory of Pharmacology, School of Basic Medicine, Jining Medical University, Jining, Shandong, China; ^2^ Department of Pathology, Second People’s Hospital of Ningyang, Taian, Shandong, China; ^3^ Department of Pathology, People’s Hospital Affiliated to Shandong First Medical University, Jinan, Shandong, China

**Keywords:** ulcerative colitis, GHK-Cu, SIRT1/STAT3 pathway, co-culture system, network pharmacology, molecular docking

## Abstract

**Introduction:**

Ulcerative colitis (UC) is a chronic inflammatory bowel disease (IBD) characterized by mucosal damage and impaired epithelial barrier function. While glycyl-l-histidyl-l-lysine-copper (GHK-Cu) exhibits known anti-inflammatory properties, its therapeutic mechanisms in UC remain undefined. This study was designed to systematically evaluate the therapeutic potential of GHK-Cu in a dextran sulfate sodium (DSS)-induced murine model of UC, with particular emphasis on elucidating its regulatory effects on the NAD-dependent deacetylase sirtuin-1 (SIRT1)/signal transducer and activator of transcription 3 (STAT3) signaling pathway.

**Methods:**

UC was induced in BALB/c mice with 3% DSS for 14 days. The protein expression levels of tight junction associated protein-1 (ZO-1), Occludin, inflammatory factors interleukin (IL)-6, IL-1β and tumor necrosis factor (TNF)-α, SIRT1, STAT3, p-STAT3, and retinoic acid receptor-related orphan receptor gamma t (RORγt) were detected by Western blot. Histopathological changes were evaluated by Hematoxylin and Eosin (H&E) and Alcian blue-periodic acid-Schiff (AB-PAS). Network pharmacology and molecular docking were used to predict the core targets of GHK Cu in the treatment of UC. An in vitro UC model was also established in mouse peritoneal macrophages (MPMs) using lipopolysaccharide (LPS), and a co culture model was constructed using mouse colonic epithelial cells (MCECs) and MPMs to examine the role of GHK Cu in promoting mucosal healing. STAT3 was silenced by gene transfection technology to verify the core role of STAT3 in GHK Cu treatment of UC.

**Results:**

GHK-Cu alleviated weight loss, improved the disease activity index (DAI), reduced colonic edema and shortening, attenuated inflammatory damage, increased goblet cell numbers, suppressed inflammatory cytokines such as TNF-α, IL-6, and IL-1β, and promoted mucosal repair. Additionally, a co-culture system of MCECs and MPMs revealed that GHK-Cu facilitated MCECs healing, impaired by DSS, by upregulating ZO-1 and Occludin expression. Subsequently, network pharmacology and molecular docking identified SIRT1 as a potential target of GHK-Cu. Results showed that GHK-Cu upregulated SIRT1 protein expression and suppressed the expression of phosphorylated p-STAT3 in colon tissue and MCECs of the co-culture model. Our findings revealed that after transfection with STAT3-targeting siRNA (siSTAT3), the stimulant effect of GHK-Cu on the healing of MCECs and the effect on the protein expression of ZO-1 and Occludin is canceled. Nevertheless, after transfection with siSTAT3, it could inhibit the expression of inflammatory factors in conjunction with GHK-Cu. Furthermore, we found that GHK-Cu could inhibit RORγt expression in the colon tissue of UC mice.

**Discussion:**

This study found that GHK-Cu demonstrated significant therapeutic effects in DSS-induced UC in mice. GHK-Cu may promote mucosal healing and enhance tight junction protein expression by regulating the SIRT1/STAT3 pathway. In addition to suppressing p-STAT3 expression, GHK-Cu may utilize additional pathways to inhibit inflammatory factors. Furthermore, GHK-Cu may reduce the number of Th17 cells. In summary, GHK-Cu may treat UC by acting on the SIRT1/STAT3 pathway.

## 1 Introduction

Ulcerative colitis (UC) is a chronic, relapsing form of inflammatory bowel disease (IBD) characterized by immune system dysfunction and pathological overactivation of mucosal immune responses, leading to persistent inflammation and subsequent colonic mucosal injury (Raine et al., 2022). Recent data indicate that UC affects approximately 5 million individuals worldwide as of 2023, with a steadily rising incidence rate (Le Berre et al., 2023). Typically, patients suffering from UC exhibit a range of clinical symptoms, which encompass abdominal pain, persistent diarrhea, rectal bleeding, as well as unintended weight loss (Akiyama et al., 2022; Krugliak Cleveland et al., 2022). The etiology of UC remains a complex puzzle, potentially involving intricate interactions among inherent genetic susceptibility, external environmental stimuli, microbial infections, disruption of the delicate balance of the gut microbiota, and abnormal immune system function (Ramos and Papadakis, 2019). Current pharmacological strategies for UC primarily include 5-aminosalicylic acid (5-ASA), glucocorticoids, thiopurines, biological medications, and anti-cytokine therapies (Lamb et al., 2019; Jori et al., 2023). However, these therapies are often limited by drug intolerance, notable side effects, high recurrence rates, and elevated costs (Ariyaratnam and Subramanian, 2014; Lamb et al., 2019; Tsujii et al., 2022). These challenges underscore the urgent need for innovative and effective drugs to overcome these shortcomings and provide more effective and tolerable treatments for UC patients.

Sirtuins (SIRTs) represent a family of evolutionarily conserved enzymes that are dependent on NAD^+^, categorized into seven distinct members (SIRT1 through SIRT7) (Wan and Garg, 2021; Ji et al., 2022). Through its deacetylase activity, the NAD^+^-dependent deacetylase sirtuin-1 (SIRT1) meticulously regulates a variety of substrates, such as histone and non-histone proteins, thereby producing significant effects on metabolic homeostasis, apoptosis regulation, inflammatory responses, attenuation of oxidative stress, and even the aging process (Tanno et al., 2007; Wu et al., 2021; Ungurianu et al., 2023). SIRT1 exhibits broad expression across intestinal epithelial cells, including the colon and small intestine, especially in the crypt region (Wellman et al., 2017). Emerging research underscores the strong correlation between SIRT1 and inflammatory processes (Wu Q. J. et al., 2022; Yang et al., 2022). Specifically, the decrease in SIRT1 levels, inhibition of its activity, or generation of mutations that lower its activity have been linked to intestinal inflammation and colitis in human and rodent models (Caruso et al., 2014; Melhem et al., 2016; Wellman et al., 2017).

Signal transducer and activator of transcription 3 (STAT3), a crucial cytoplasmic transcription factor in the STAT family, consists of 770 amino acids and possesses a complex domain structure, facilitating its diverse roles in cell signaling and gene regulation (Ma et al., 2020). By regulating the transcription of specific genes, STAT3 significantly impacts various biological processes such as cell growth, differentiation, metabolic activities, angiogenesis, and immune responses (Zou et al., 2020; Li et al., 2023). By upregulating key genes such as interleukin-17 (IL-17) and retinoic acid receptor-related orphan receptor gamma t (RORγt), which are essential for Th17 cell differentiation and inflammation regulation, STAT3 effectively amplifies the inflammatory cascade reaction (Atreya et al., 2000; Parihar and Bhatt, 2023). Extensive research has unequivocally highlighted the pivotal role of Th17 cells in the pathogenesis of UC (Fitzpatrick et al., 2020; Lv et al., 2023). Additional research indicated that the proper function of STAT3 is critical for maintaining intestinal homeostasis, and constitutive activation of the STAT3 signaling pathway in epithelial cells reduces tight junction protein expression within endothelial cells, consequently enhancing vascular permeability and contributing to tissue damage and disease progression (Spalinger et al., 2021). It is noteworthy that clinical studies have revealed an upregulation in the expression of STAT3 within the intestinal mucosa of individuals suffering from IBD (Li et al., 2010). In summary, targeting STAT3 and its related pathways may help prevent the occurrence and progression of inflammatory diseases (Zhao et al., 2021). Histone deacetylases including SIRT1, interact with STAT3 and negatively regulate its transcriptional activity by deacetylating critical lysine residues (Nie et al., 2009). This delicate regulatory mechanism highlights the essential role of SIRT1 in maintaining balance within complex cell signaling networks.

**FIGURE 1:**
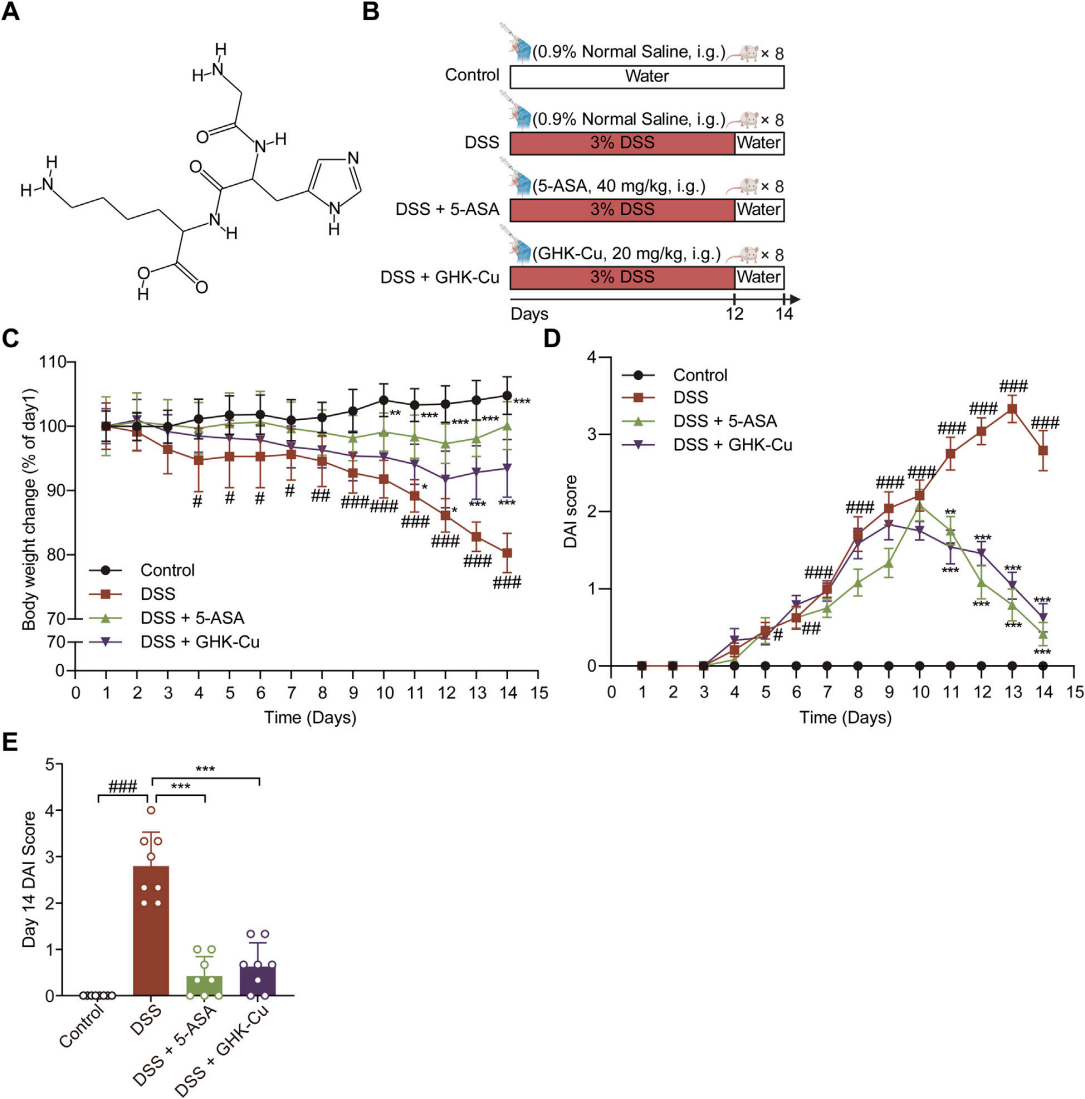
GHK-Cu could alleviate DSS-induced colitis. **(A)** Chemical structure of GHK. **(B)** Experimental scheme of the animal experimental design. **(C)** Body weight changes of all groups. **(D)** DAI score of all groups. **(E)** DAI scores for day 14. ^#^ *P* < 0.05, ^##^ *P* < 0.01, ^###^ *P* < 0.001 compared with Control group; ^*^ *P* < 0.05, ^**^ *P* < 0.01, ^***^ *P* < 0.001 compared with DSS group.

Short peptides are distinctive biomolecules that combine the benefits of traditional small molecules and mature proteins, offering new, more effective therapeutic agents with fewer side effects (Apostolopoulos et al., 2021; Bojarska, 2022). Glycyl-L-histidyl-L-lysine (GHK) is a peptide found naturally in plasma, saliva, and urine, and its concentration tends to diminish with increasing age (Dou et al., 2020). GHK exhibits a Cu^2+^ affinity comparable to that of albumin’s copper transport sites, forming an *in vivo* complex, glycyl-L-histidyl-L-lysine-Cu^2+^(GHK-Cu), which enhances its bioavailability (Pickart, 2008). The chemical structure of GHK is depicted in Figure 1A. Over the past several decades, the efficacy of GHK and GHK-Cu in promoting tissue remodelling, exhibiting anti-inflammatory properties, and demonstrating antioxidant effects has been robustly validated across diverse tissues, encompassing the skin (Pickart et al., 2015), airways (Zhang et al., 2023), bones (Klontzas et al., 2019), skeletal muscles (Deng et al., 2023), stomach (Alberghina et al., 1992), and lungs (Zhang et al., 2022). Similarly, GHK-Cu has demonstrated the ability to suppress the progression of ulcers in intestinal models (Pickart, 2008). However, the therapeutic effects of GHK-Cu on UC have not been fully investigated. It is reported that 5-ASA ameliorates UC in mice through dual mechanisms of preserving intestinal epithelial barrier integrity and modulating gut microbiota composition, with concomitant suprpression of JNK and p38 MAPK phosphorylation in colonic macrophages (Qu et al., 2017; Wang et al., 2025). Based on the aforementioned effects and mechanisms of 5-ASA, we employed 5-ASA as the positive control drug in our *in vivo* experiments. This study aimed to investigate the therapeutic effect of exogenous GHK-Cu on UC mice and explore its mechanism.

## 2 Materials and methods

### 2.1 Reagents

Dextran sulfate sodium salt (DSS, MW 36,000–50,000 Da, batch number 160110) was purchased from MP Biomedicals. 5-Aminosalicylic acid (5-ASA, batch number A129982) was purchased from Aladdin. Fecal occult blood qualitative test kit (Pyridinium chlorochromate method, batch number R24187) was obtained from Shanghai Yuanye. Lipopolysaccharide (LPS, batch number L6529) was acquired from Sigma. MTT Cell Proliferation and Cytotoxicity Assay Kit (batch number C0009S) was obtained from Beyotime. Thiolglycollate medium (batch number LA0561), red blood cell lysis buffer (batch number R1010), and dimethyl sulfoxide (DMSO, batch number D8371) were purchased from Solarbio. Occludin monoclonal antibody (batch number 66378) and STAT3 polyclonal antibody (batch number 10253-2-AP) were obtained from Proteintech. Zonula occludens-1 (ZO-1, batch number AF5145), Beta-Actin antibody (batch number AF7018), Goat Anti-Mouse IgG (H + L) HRP (batch number S0002), and Goat Anti-Rabbit IgG (H + L) HRP (batch number S0001) were obtained from Affinity. Rabbit Anti-TNF alpha Polyclonal Antibody (batch number 2081R) and Rabbit Anti-IL-6 Polyclonal Antibody (batch number 0782R) were obtained from Bioss. Anti-IL-1 beta Rabbit pAb (batch number GB11113) was purchased from Servicebio. Anti-SIRT1 antibody (batch number 189494) and Anti-ROR gamma antibody (batch number 207082) were obtained from Abcam. Rabbit monoclonal antibody Phospho-Stat3 (Tyr705, batch number 91455) was purchased from Cell Signaling Technology. Transwell permeable supports (batch number 3412) were purchased from Corning. Lipofectamine 2000 (batch number 11668500) was purchased from Thermo Fisher. STAT3-targeting siRNA (siSTAT3, batch number 1348C5) was purchased from GeneChem.

### 2.2 Animals

Thirty-two male BALB/c mice, aged 6–8 weeks and weighing 18–22 g, were provided by Jinan Pengyue Experimental Animal Breeding Co., Ltd. During the entire experiment, the mice were housed under rigorously controlled conditions, including a temperature of 23°C ± 2°C, a relative humidity of 45% ± 5%, and a consistent 12-hour light/dark cycle. Additionally, they were provided with unrestricted access to food and water to ensure their wellbeing. The experimental protocol was approved by the Institutional Animal Care and Use Committee of Jining Medical University (Approval No. JNMC-2022-DW-162).

### 2.3 DSS-induced models and GHK-Cu treatment

DSS is extensively utilized in animal models of UC due to its capacity to induce histopathological, clinical, and immunologic responses that closely resemble the manifestations observed in patients with IBD (Eichele and Kharbanda, 2017). Following a 1-week acclimatization period, the mice were randomly assigned to four groups (n = 8): Control, DSS, DSS + 5-ASA, and DSS + GHK-Cu. In all groups aside from the Control, a 3% DSS solution was prepared using distilled water and administered as drinking water for 12 days, followed by a return to standard drinking water for the subsequent 2 days. Mice in the Control and DSS groups received daily saline gavage for 14 days, while the treatment groups were gavaged with either 5-ASA (40 mg/kg) or GHK-Cu (20 mg/kg). All mice were euthanized on day 15.

### 2.4 Evaluation of colitis

Throughout the animal experiment, the mental state, stool consistency, fur quality, perianal condition, and fecal occult blood of the experimental mice were assessed daily at a fixed time. The progression of colitis was monitored using the Disease Activity Index (DAI) scoring system (Yao et al., 2020), which assesses the severity of colitis based on specific parameters. The scoring criteria for DAI are presented in Table 1.

**TABLE 1:** The scoring criteria of DAI.

Score	Weight loss (%)	Stool consistency	Fecal occult blood
0	0	Normal	Negative
1	1–5	—	Light blue
2	5–10	Loose Stool	Blue
3	10–15	—	Dark blue
4	≥15	Diarrhea	Gross blood

### 2.5 Harvesting of primary organs and colon tissues

On the 15th day of the experiment, euthanasia was carried out on all the mice. Colon segments were promptly removed, carefully straightened to their natural length, and measured. After longitudinal dissection along the mesentery, the colon was rinsed thoroughly with pre-cooled saline and blotted dry with filter paper. The severity of colon mucosal damage was evaluated using the colonic mucosal damage index (CMDI) following the criteria outlined by Jiang et al. (2021). A score of 0 indicates no inflammation or ulcers, while a score of 1 reflects localized hyperemia in the absence of ulcers. A score of 2 represents hyperemia accompanied by thickening of the intestinal wall without ulcers. A score of 3 denotes one ulcer with inflammation measuring approximately 0–1 cm in diameter. A score of 4 is assigned to cases involving two or more ulcers with associated inflammation, measuring 1.1–2 cm in diameter, without adhesion to adjacent organs. Finally, a score of 5 corresponds to ulcers extending beyond 2 cm, accompanied by marked intestinal thickening and severe adhesion to surrounding organs. Colon tissues with visible lesions were carefully fixed in 10% neutral buffered formalin for subsequent hematoxylin-eosin (H&E) and alcian blue-periodic acid-Schiff (AB-PAS) staining. The remaining colon samples were stored at −80°C for future protein blot analysis. The heart, liver, spleen, lungs, and kidneys were carefully harvested and weighed.

### 2.6 H&E and AB-PAS staining

Paraffin-embedded colonic tissue blocks were cut into 4-µm-thick sections for H&E staining (Duan et al., 2023). The histological change scoring criteria are shown in Table 2. Paraffin sections were stained using the AB-PAS method and then examined and quantified for goblet cells under a microscope (Xu et al., 2023).

**TABLE 2:** Scoring criteria for histological changes.

Score	Number of ulcers	Epithelial changes	Lesion depth
0	0	Normal	Normal
1	1	Goblet cell loss	Mucous membrane
2	2	Massive goblet cell loss	Submucosa
3	3	Crypt deletion	Muscle
4	4	Extensive deletion of crypts	Serosa

### 2.7 Network pharmacology

Compound information was obtained through PubChem. This information was then utilized to retrieve the corresponding targets of GHK through the SwissTargetPrediction and BATMAN-TCM databases. After removing duplicates, UniProt standardized entries were used to identify drug-relevant targets. Genes associated with UC were collected using the keyword “ulcerative colitis” from five databases: GeneCards, DrugBank, Online Mendelian Inheritance in Man (OMIM), PharmGKB, and TTD. After deduplication and standardization, the final UC-related targets were obtained. The collected targets were imported into Venny 2.1 to acquire “drug-disease” targets. Protein-protein interaction (PPI) data originated from the STRING database, and Cytoscape software was employed for visualization. The CentiScape 2.2 plugin facilitated topological analysis of the targets, and the results were visualized as a network graph. Different nodes represented distinct target proteins, and connections depicted their interaction relationships. Node degree was one of the primary methods used to identify key nodes, with higher node degrees signifying greater importance in the network.

### 2.8 Molecular docking

The 3D structure for GHK-Cu was retrieved from PubChem and converted into “mol2” format with Open Babel 3.1.1. The small molecule ligand was processed using AutoDockTools 1.5.7 software, including adding hydrogen bonds and assigning proper torsion angles to chemical bonds, and then exported in PDBQT format. A search for the macromolecular structure of SIRT1 was conducted within the PDB database, and the file was retrieved in PDB format. The macromolecular receptor was processed using AutoDockTools 1.5.7 software, including the addition of hydrogen bonds, calculation of charges, setting of rigid structures, and designation as the receptor, and then exported in PDBQT format. A box was constructed to fully enclose the small molecule and protein by adjusting the “Grid Box” parameters, followed by running AutoGrid 4, setting the docking parameters and algorithms, running AutoDock4, and exporting the results file in PDBQT format. The docking results were visualized using PyMOL 2.4.1 software. Lower binding energy between the macromolecular protein and small molecule ligand indicates a higher binding probability and stability.

### 2.9 Cell culture and treatment

Mouse colon epithelial cells (MCECs) were cultured in high-glucose Dulbecco’s Modified Eagle Medium, supplemented with 10% fetal bovine serum and 1% penicillin-streptomycin (P/S), in a humidified incubator with 5% CO_2_ at 37°C.

Mice were intraperitoneally injected with 3% thiolglycollate medium, and mouse peritoneal macrophages (MPMs) were collected after 72 h. Subsequently, the cells were seeded in a 6-well plate at a density of 3 × 10^6^ cells per well. Culturing was performed in RPMI 1640 medium enriched with 10% fetal bovine serum, maintained at 37°C in a humidified incubator with an atmosphere of 5% CO_2_ and 95% air. Prior to stimulating the cells with LPS (1 mg/mL), they were pretreated with GHK-Cu or left untreated for 18 h. Proteins were then extracted and stored at −20°C for future analysis.

### 2.10 Cell viability assays

MCECs and MPMs were seeded at densities of 1 × 10^4^ and 5 × 10^4^ cells/100 μL, respectively, in a 96-well plate. After cell adhesion, the media containing different concentrations of compounds were added and incubated for 24 h. The MTT assay was performed according to the instructions, and absorbance was measured at 490 nm.

### 2.11 Co-culture and wound healing assay

MCECs were seeded into 6-well plates and cultured for 24 h. MPMs were seeded in the upper chamber of the Transwell system with a pore size of 0.4 µm and co-cultured with MCECs in the 6-well plate, followed by treatment with GHK-Cu (62.5 µM). After 24-hour treatment with GHK-Cu, the width of the scratch was measured, photographed, and quantitatively evaluated.

### 2.12 Cell transfection

MCECs or MPMs were seeded in 6-well plates and transfected with siSTAT3 or the corresponding negative controls using Lipofectamine 2000 reagent. The transfection protocol was consistent with previous reports (Chen et al., 2024).

### 2.13 Western blot

Proteins were extracted from colon tissue and cell samples and stored at −20°C. The expression of ZO-1, Occludin, SIRT1, STAT3, p-STAT3, RORγt, IL-6, IL-1β, TNF-α, and β-actin was examined using the method previously described (Yao et al., 2014).

### 2.14 Statistical analysis

Data are presented as the mean ± standard deviation. Statistical analysis was performed using IBM SPSS version 26.0 (IBM Corporation, Chicago, United States). One-way ANOVA was used to compare the mean values across multiple groups, followed by Dunnett’s test for *post hoc* multiple comparisons to allowing for a detailed examination of significant differences between different experimental groups. *P* values less than 0.05 were considered statistically significant.

## 3 Results

### 3.1 GHK-Cu could alleviate DSS-induced colitis

**FIGURE 2:**
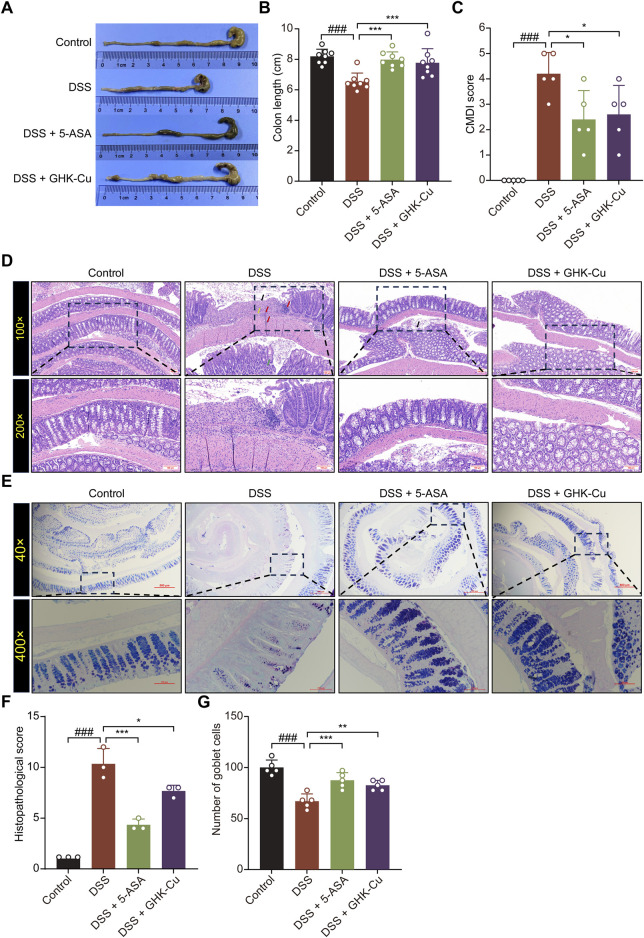
GHK-Cu could reduce pathological damage induced by DSS. **(A,B)** The macroscopic representations and the length of colons. **(C)** CMDI score. **(D)** H&E staining of colon sections (100×, 200×). Red arrows indicated inflammatory cell infiltration; black arrows indicated necrotic and ulcerated areas; green arrows pointed to structural damage within the colonic crypts; yellow arrows indicated colon edema and fibrosis. **(E)** AB-PAS staining of colon sections (200×, 400×). **(F)** Pathological scores of all groups. **(G)** Goblet cell counts of all groups. ^###^ *P* < 0.001 compared with Control group; ^*^ *P* < 0.05, ^**^ *P* < 0.01, ^***^ *P* < 0.001 compared with DSS group.

The experimental protocol for the animals is shown in Figure 1B. DSS-induced colitis in mice elicited pronounced symptoms, including reduced locomotor activity, decreased consumption of food and water, dull and lifeless fur, signs of depression-like behavior, huddling behavior, perianal swelling and congestion, diarrhea, bloody stools, and substantial weight loss. GHK-Cu treatment alleviated these symptoms. As illustrated in Figure 1C, the body weight of mice in the DSS group underwent a significant decrease. However, the administration of GHK-Cu reversed this downward trend by day 12. In addition, compared to the Control group, the DAI score in the DSS group exhibited a sharp increase starting on day 4. Conversely, treatment with GHK-Cu led to a significant reduction in this score (Figures 1D,E). As evident from Figures 2A,B, the colon length of mice with DSS-induced colitis was notably shorter than that of the Control group. GHK-Cu treatment significantly mitigated this reduction in colon length. The CMDI, serving as a macroscopic assessment of extensive injury in the specimens, was markedly increased in the DSS group. Following treatment with GHK-Cu, this index was significantly reduced (Figure 2C). Histopathological examination using H&E and AB-PAS staining revealed severe pathological changes in the colonic epithelium of DSS-treated mice. These changes included mucosal erosion, ulcerative lesions, deformation and distortion of colonic recesses, extensive infiltration of inflammatory cells (primarily lymphocytes and neutrophils) in the mucosa and submucosa, and depletion of goblet cells. Nevertheless, after intervention with GHK-Cu, a significant reduction in these pathological changes was observed (Figures 2D–G). These findings suggested that GHK-Cu had therapeutic effects in mitigating the inflammatory pathological damage in mice with UC.

### 3.2 GHK-Cu inhibited the expression of inflammatory cytokines in DSS-induced colitis mice

**FIGURE 3:**
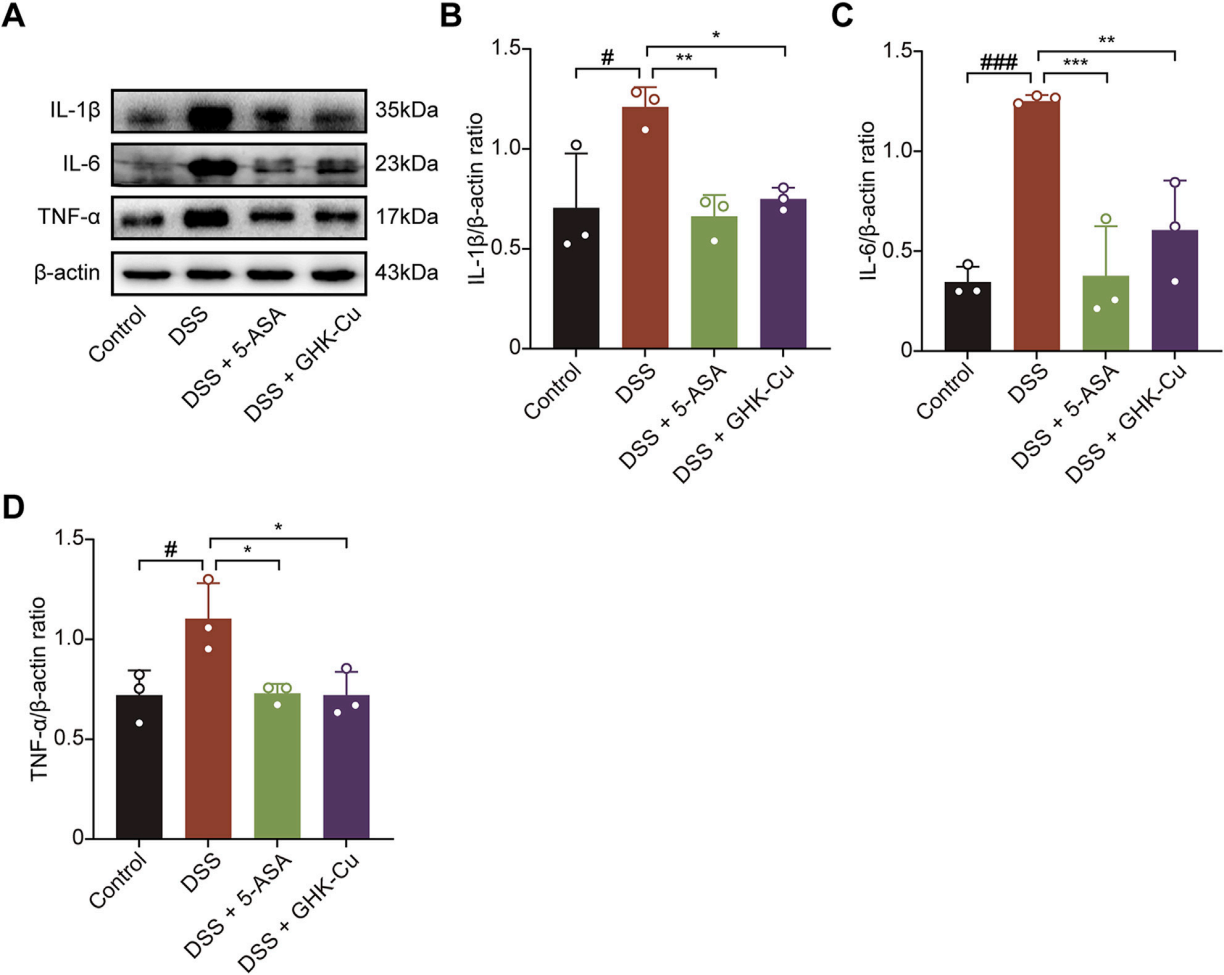
GHK-Cu inhibited the expression of inflammatory cytokines in DSS-induced mice. **(A–D)** Western blot analysis of the expression of IL-1β, IL-6 and TNF-α in colonic tissues of all groups of mice. ^#^ *P* < 0.05, ^###^ *P* < 0.001 compared with Control group; ^*^ *P* < 0.05, ^**^ *P* < 0.01, ^***^ *P* < 0.001 compared with DSS group.

Figures 3A–D show that Western blot analysis detected marked elevations in IL-1β, IL-6, and TNF-α levels in colonic tissue of DSS-induced colitis mice, indicating a robust inflammatory response. Moreover, GHK-Cu treatment significantly reduced the expression of these pro-inflammatory cytokines.

### 3.3 GHK-Cu promoted mucosal healing

**FIGURE 4:**
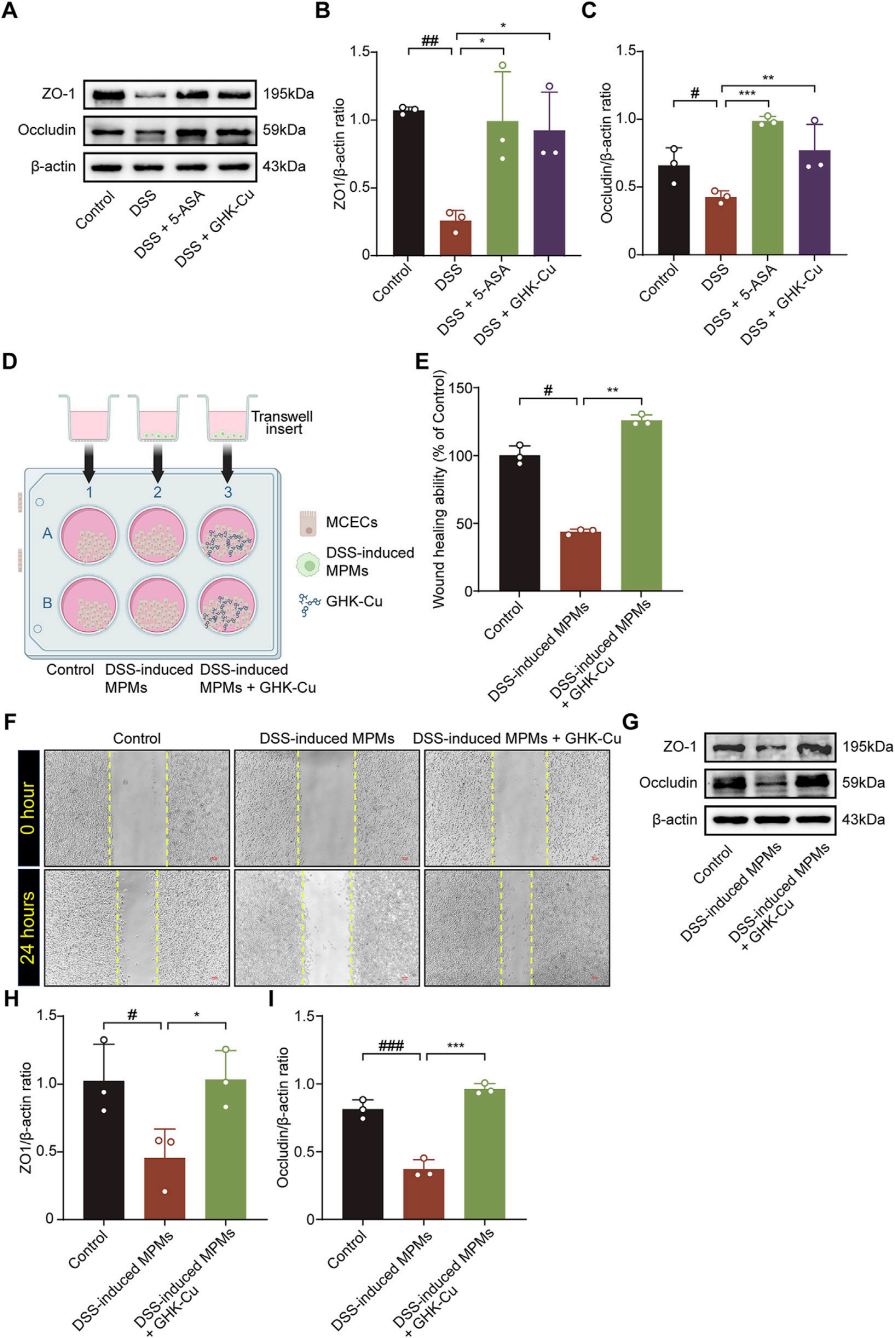
GHK-Cu contributed to mucosal healing. **(A–C)** Western blot analysis of the protein expression of ZO-1 and Occludin in colonic tissues of different groups of mice. ^#^ *P* < 0.05, ^##^ *P* < 0.01, compared with Control group; ^*^ *P* < 0.05, ^**^ *P* < 0.01, ^***^ *P* < 0.01 compared with DSS group. **(D)** Schematic representation of a co-culture system established using by MPMs and MCECs. The elements in the graph are derived from BioRender. **(E,F)** The effect of GHK-Cu on the migration of MCECs in the co-culture system. Scale bar = 100 μm. **(G–I)** Western blot analysis of ZO-1 and Occludin expression of MCECs in the co-culture system. ^#^ *P* < 0.05, ^##^ *P* < 0.01, ^###^ *P* < 0.001 compared with Control group; ^*^ *P* < 0.05, ^**^ *P* < 0.01, ^***^ *P* < 0.001 compared with DSS-induced MPMs group.

GHK-Cu treatment significantly enhanced of ZO-1 and Occludin expression in colitic mice, which were downregulated by DSS (Figures 4A–C).

In the MTT assay, treatment with 62.5 μmol/L GHK-Cu for 24 h did not significantly reduce cell viability (*P* > 0.05, data not shown). The co-culture system established with MCECs and MPMs is shown in Figure 4D. DSS-induced MPMs impaired MCECs migration, while GHK-Cu augmented this capacity (Figures 4E,F). In the co-culture system, MCECs derived from the model group exhibited significant reduced ZO-1 and Occludin expression compared to those from the Control group, whereas GHK-Cu elevated theses protein levels (Figures 4G–I).

### 3.4 The target network and molecular docking with SIRT1 of GHK-Cu

**FIGURE 5:**
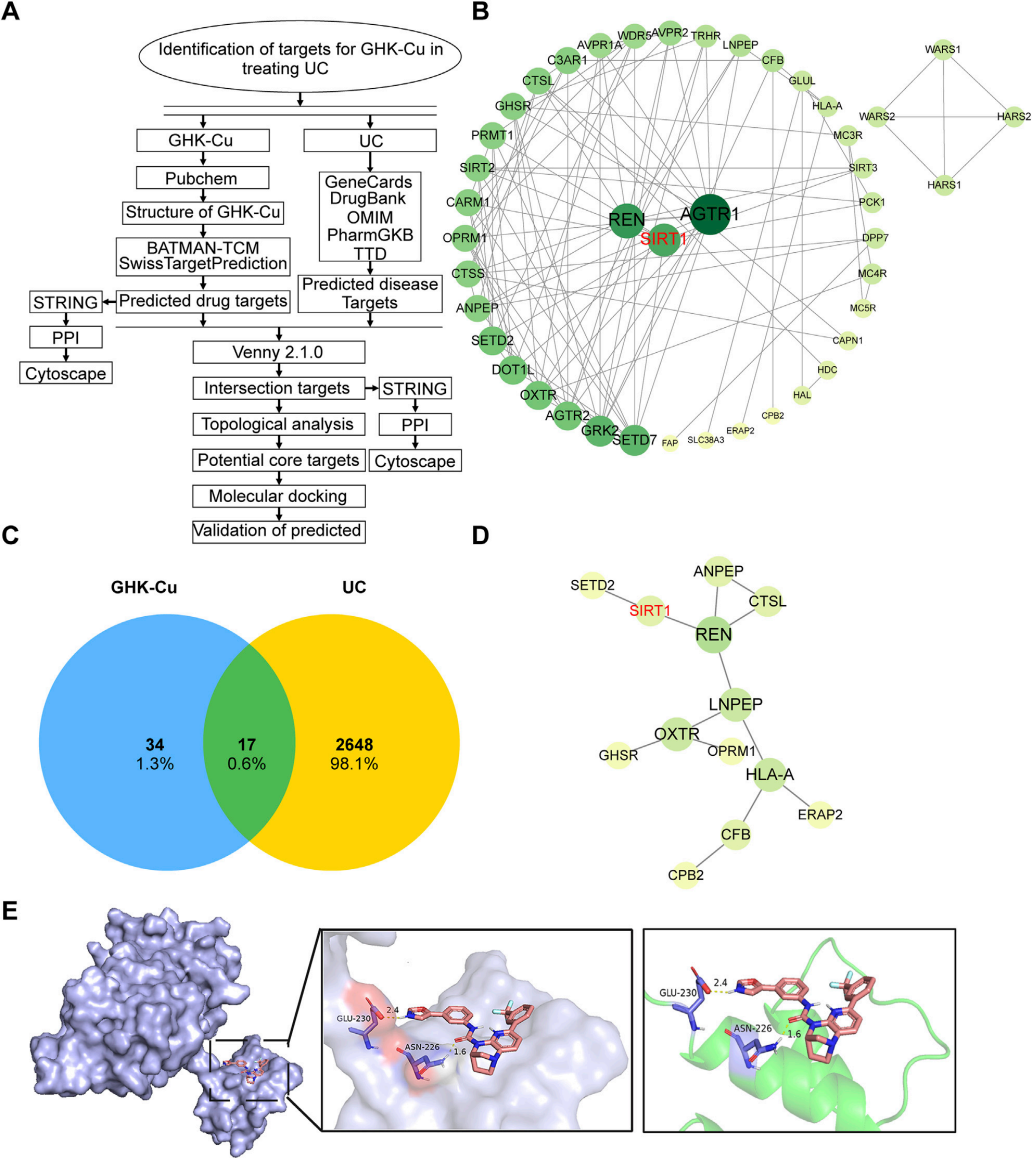
The target network and molecular docking with SIRT1 of GHK-Cu. **(A)** A flowchart outlining the network pharmacology-based drug target screening process. **(B)** PPI network map of GHK-Cu-related targets. **(C)** Common target of UC and GHK-Cu **(D)** PPI interaction network of the cross targets of GHK-Cu and UC **(E)** Molecular docking diagram of GHK-Cu and target SIRT1.

A flowchart of the drug target screening based on network pharmacology is shown in Figure 5A. Obtain the structure (Figure 1A) and the identification number (342,538) of GHK from PubChem. Then, query two commonly used target prediction databases, SwissTargetPrediction and Batman-TCM, to obtain 41 and 11 potential targets, respectively. After eliminating duplicates, 51 targets with standardized names were acquired. Cytoscape 3.9.1 software was utilized to construct the network of interactions illustrated in Figure 5B, which shows the connections between GHK-Cu and its targets. The targets are sorted based on their importance values. The color and size of each node in the network increase proportionally with higher degree values.

Using “ulcerative colitis” as the search keyword, we screened five databases related to UC-associated genes. After removing duplicates entries and standardizing the data, 2,665 targets were obtained. The interactions between drugs and disease targets were visualized using Venny 2.1 to generate a Venn diagram, revealing 17 drug-disease targets (Figure 5C). Subsequently, the 17 identified drug-disease targets were uploaded to the STRING database. After filtering out disconnected nodes, a protein-protein interaction (PPI) network comprising 17 nodes and 13 edges was obtained. The topological properties of the drug-disease network were analyzed using Cytoscape software, as demonstrated in Figure 5D. By integrating the network graph and the topological attributes table, we identified five key targets associated with UC, including REN, LNPEP, HLA-A, OXTR, and SIRT1. These targets are crucial to the therapeutic efficacy of GHK-Cu in treating UC. Based on previous studies and a review of relevant literature, we hypothesized that SIRT1 is a promising target for GHK-Cu in UC treatment, which will be verified in the subsequent experiments.

Next, molecular docking analysis was conducted to investigate whether GHK-Cu directly interacts with SIRT1. Among the 50 generated conformations, the optimal docking result was selected based on Gibbs free energy, and further downstream analysis was focused on this highly ranked conformation. The selected complex exhibited a binding energy of −8.75 kcal/mol. The simulated results of molecular docking indicated that GHK-Cu can directly bind to SIRT1 to form a protein complex, and the interacting residues in the complex are composed of GLU-230 and ASN-226 (Figure 5E).

### 3.5 GHK-Cu regulated the SIRT1/STAT3 pathway

**FIGURE 6:**
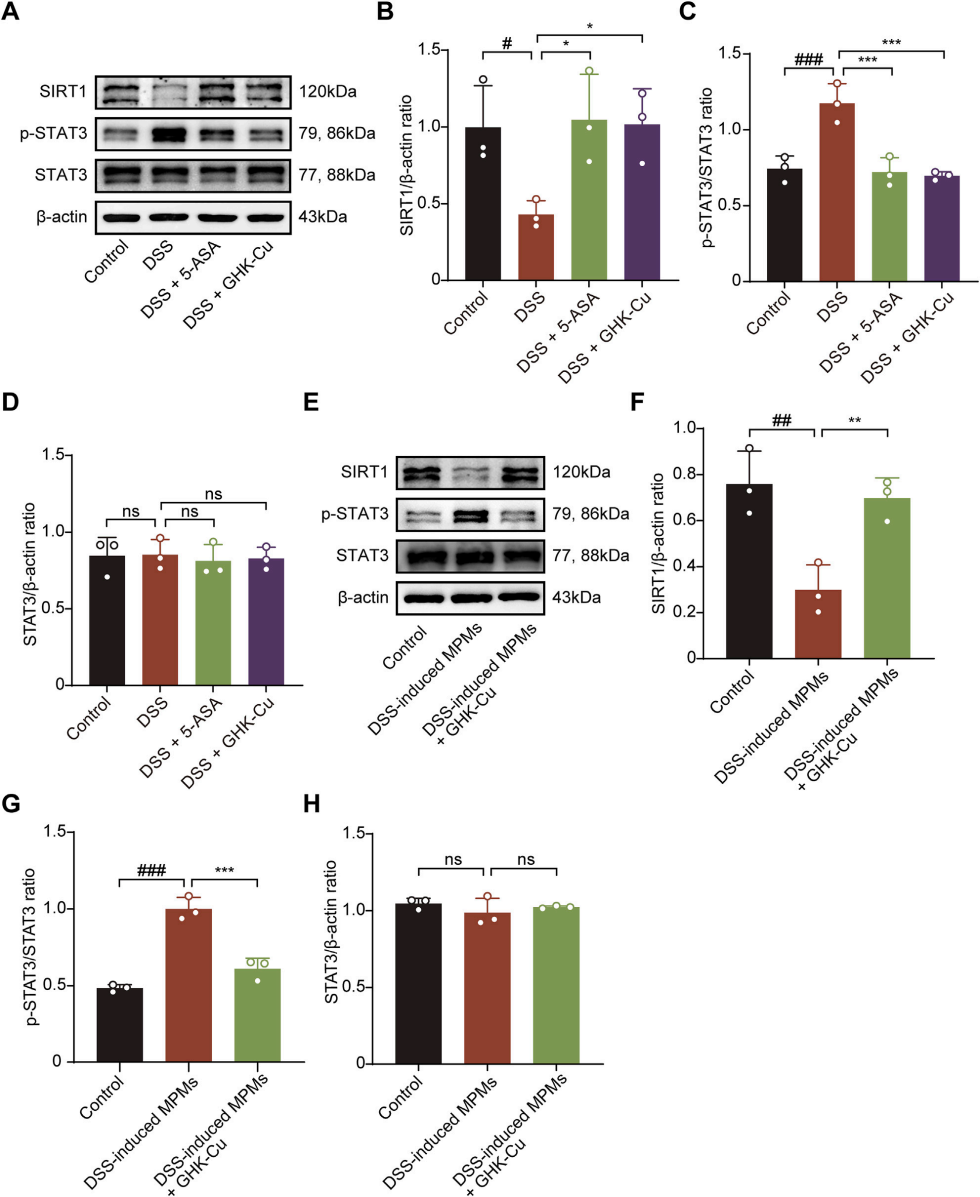
GHK-Cu modulated the expression levels of SIRT1 and p-STAT3 in colon tissues and MCECs within co-culture systems. **(A–D)** Western blot analysis of SIRT1, p-STAT3 and STAT3 protein expression in colonic tissues of different groups of mice. ^#^ *P* < 0.05, ^##^ *P* < 0.01 compared with the Control group; ^*^ *P* < 0.05, ^***^ *P* < 0.001 compared with DSS group. **(E–H)** Western blot analysis of SIRT1, p-STAT3 and STAT3 protein expression of MCECs in co-culture system. ^##^ *P* < 0.01, ^###^ *P* < 0.001 compared with the Control group; ^**^ *P* < 0.01, ^***^ *P* < 0.001 compared with DSS-induced MPMs group.

SIRT1, which is closely associated with cellular metabolism and stress responses, also interacts with the STAT3 pathway (Wu Q. J. et al., 2022; Yang et al., 2022; Huang et al., 2024), modulating the inflammatory process in colitis. As shown in Figures 6A–D, DSS-induced mice exhibited significantly increased SIRT1 expression and decreased p-STAT3 expression compared to the Control group. In contrast, GHK-Cu treatment reversed these alterations. Similarly, the expression levels of these proteins followed the same trend in co-cultured MCECs (Figures 6E–H).

### 3.6 GHK-Cu promoted mucosal healing via inhibiting STAT3 activation

**FIGURE 7:**
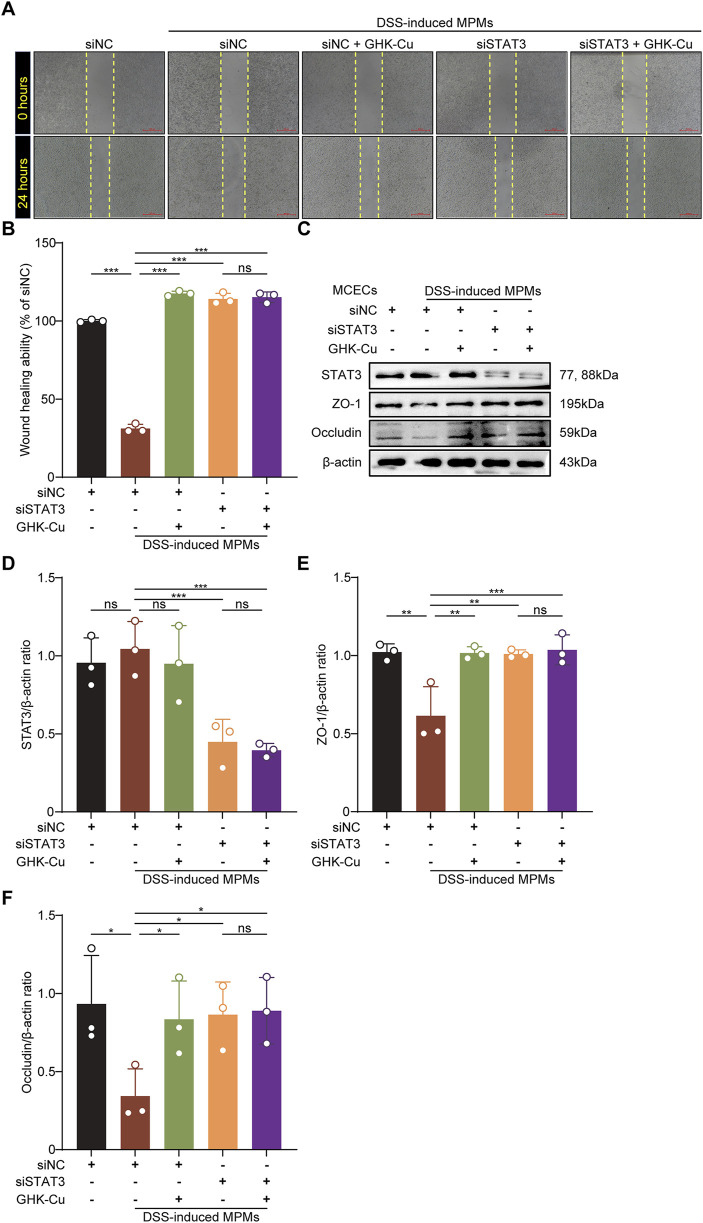
GHK-Cu promoted mucosal healing by inhibiting the activation of STAT3. The MCECs within the co-culture system were stimulated with DSS induced MPMs and transfected with STAT3-targeting siRNA (siSTAT3)/nontargeting siRNAs (siNC), with or without GHK-Cu pretreatment (62.5 µM). **(A,B)** The effect of GHK-Cu on the migration of MCECs in the co-culture system. Scale bar = 100 μm. **(C–F)** Western blot analysis of STAT3, ZO-1 and Occludin expression of MCECs in the co-culture system. All data are presented as mean ± SD with n = 3 per group. ^*^ *P* < 0.05, ^**^ *P* < 0.01, ^***^ *P* < 0.001.

To investigate the role of STAT3 activation in facilitating the therapeutic benefits of GHK-Cu, we meticulously designed and executed STAT3 knockdown experiments *in vitro*, utilizing the co-culture model. In STAT3-knockdown MCECs (siSTAT3-transfected), GHK-Cu failed to enhance wound healing in scratch assays (Figures 7A,B) and did not upregulate ZO-1/Occludin expression (Figures 7C–F).

### 3.7 GHK-Cu suppresses inflammatory cytokines partly via STAT3 inhibition

**FIGURE 8:**
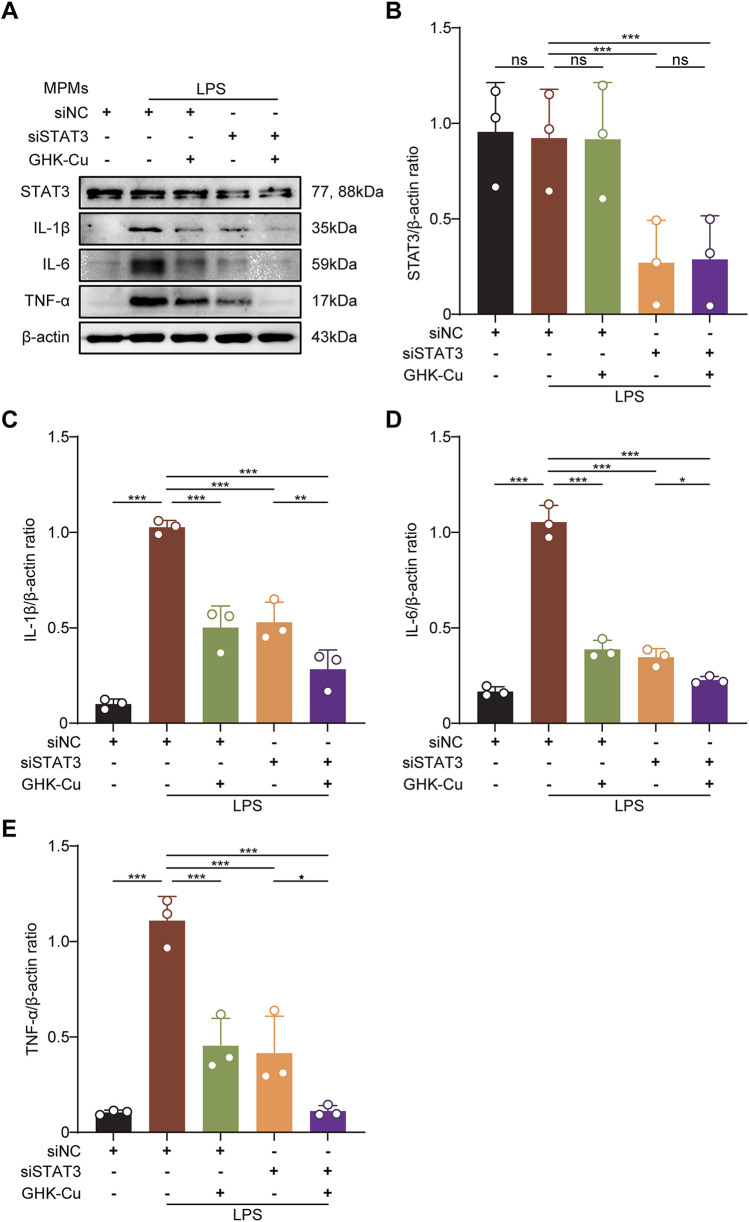
GHK-Cu inhibitd the expression of inflammatory cytokines by inhibiting the activation of STAT3. The MPMs was stimulated with LPS (1 μg/mL) and transfected with siSTAT3/siNC, with or without GHK-Cu pretreatment (62.5 µM). **(A–E)** Western blot analysis of the expression of STAT3, IL-1β, IL-6 and TNF-α in MPMs of all groups. All data are presented as mean ± SD with n = 3 per group. ^*^ *P* < 0.05, ^**^ *P* < 0.01, ^***^ *P* < 0.001.

We also explored the role of STAT3 in modulating inflammatory factor expression by GHK-Cu in LPS-stimulated MPMs transfected with siSTAT3. The results showed that after transfection with siSTAT3, GHK-Cu treatment showed a synergistic effect on the inhibition of inflammatory factors (Figures 8A–E). In other words, in addition to inhibiting the expression of p-STAT3, GHK-Cu likely targets other pathways that inhibit the expression of inflammatory factors.

### 3.8 GHK-Cu inhibited the expression of RORγt in DSS-induced colitis

**FIGURE 9:**
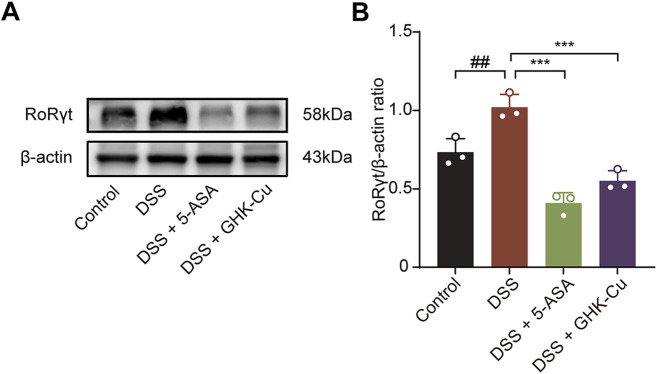
GHK-Cu inhibited the expression of RORrt in DSS-treated mice. **(A,B)** Western blot analysis of RORγt protein expression in colonic tissues of different groups of mice. ^##^ *P* < 0.01 compared with the Control group; ^***^ *P* < 0.001 compared with DSS group.

Western blot analysis showed that treatment with GHK-Cu significantly reduced DSS-induced RORγt expression (Figure 9), implying Th17 cell inhibition contributes to its therapeutic effect.

## 4 Discussion

GHK and its bioactive form, GHK-Cu, have been demonstrated to be safe and effective in various cellular, tissue, and animal models (Pickart and Margolina, 2018). GHK exhibits high affinity for copper and can spontaneously bind with it to form the chelate complex GHK-Cu (Dou et al., 2020). Notably, free copper is abundant in the human body, while other copper-binding proteins are scarce. This suggests that GHK likely exists predominantly as GHK-Cu under normal physiological conditions (Pickart, 2008). Additionally, the GHK-Cu complex has been shown to enhance GHK’s bioavailability, particularly in terms of its antioxidant and anti-inflammatory properties (Dou et al., 2020; Deng et al., 2023). Nevertheless, the current understanding of GHK and GHK-Cu’s role in digestive diseases remains limited. A pilot study involving 16 patients with distal IBD reported a mean 60% reduction in disease severity after 12 weeks of rectal GHK-Cu treatment, as assessed by endoscopic, histopathological, and symptomatic evaluations (Pickart, 2008). Consequently, this study utilized the DSS-induced UC model to investigate the therapeutic efficacy and mechanisms of action of GHK-Cu. Our findings indicated that exogenous GHK-Cu supplemention alleviated DSS-induced colitis symptoms, including decreased DAI score, mitigated colonic injury, improved histopathological scores, preserved goblet cell morphology, and increased goblet cell numbers. These results suggest that GHK-Cu may alleviate the clinical symptoms of UC, emphasizing its potential as a therapeutic agent for colitis.

In IBD, the expression levels of tight junction proteins such as ZO-1 and Occludin, is generally suppressed, leading to increased intestinal permeability, enhanced pathogen infiltration, and exacerbated inflammatory responses (Dong et al., 2022). Furthermore, findings from our *in vivo and in vitro* experimental models revealed that GHK-Cu upregulates the expression level of Occludin and ZO-1, suggesting its potential role in promoting intestinal barrier repair.

Nonetheless, the precise molecular target of GHK-Cu has not been identified. Our study preliminarily predicted that SIRT1 may be a core target of GHK in the treatment of UC by utilizing network pharmacology and molecular docking. Numerous studies have proposed that GHK-Cu acts as a potential SIRT1 activator, thereby highlighting its multifaceted role in cellular processes, especially its capacity to enhance SIRT1 activity (Deng et al., 2023; Zhang et al., 2023). SIRT1, a crucial deacetylase, is involved in diverse cellular processes, including metabolism, stress resistance, inflammation, and aging (Wu et al., 2021). Decreased SIRT1 expression in colon epithelial cells of UC patients and DSS-induced mice correlates with immune dysregulation (Caruso et al., 2014; Li et al., 2022). Consequently, SIRT1 activation represents a promising therapeutic strategy for mitigating DSS-induced UC. To validate this mechanism, we conducted in *in vivo* and *in vitro* experiments. Western blot results showed that GHK-Cu significantly upregulated SIRT1 expression in the colon tissues of DSS-induced mice and MCECs in the co-culture system. Thus, we demonstrated that GHK-Cu is a novel potential SIRT1 activator.

**FIGURE 10:**
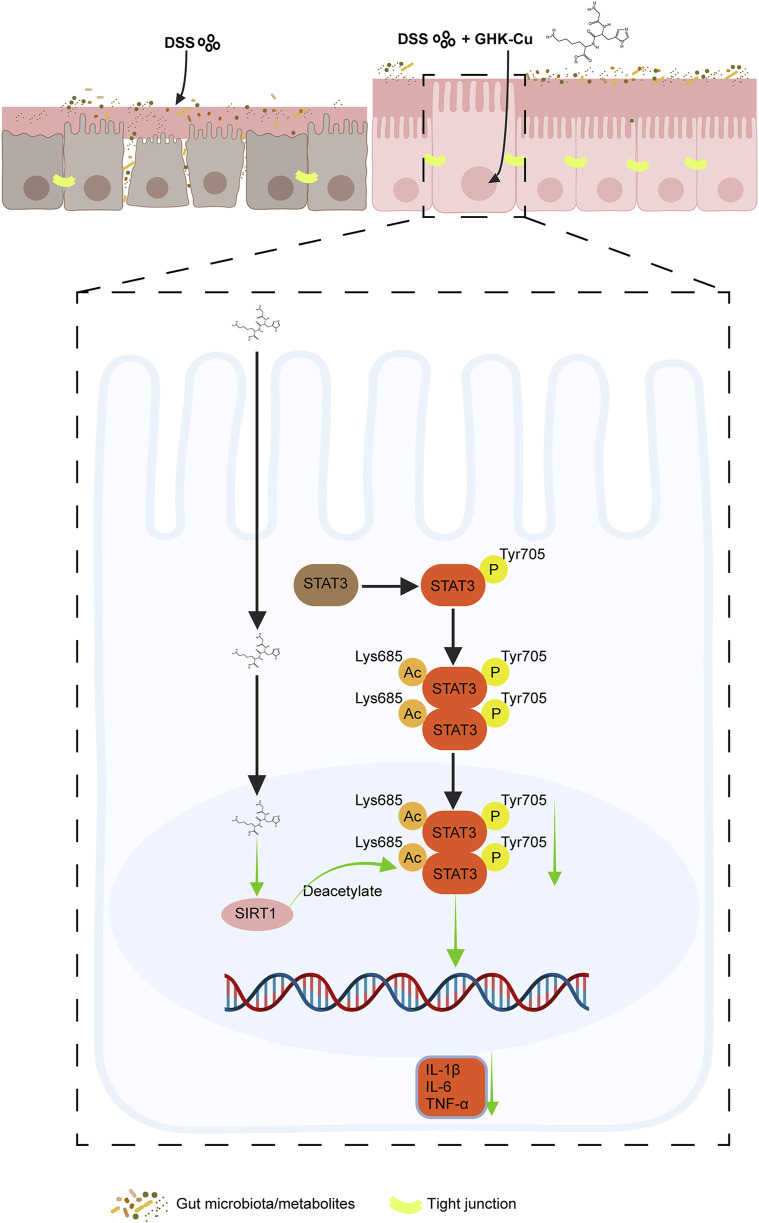
Schematic diagram of the therapeutic effect of GHK-Cu on UC and its mechanism.

STAT3 is a crucial factor in maintaining cell proliferation and survival, and can be activated by various cytokines to mediate immune and inflammatory responses (Wu X. et al., 2022). A proteome-wide Mendelian randomization analysis linked STAT3 to an increased UC risk (Chen J. et al., 2023). In colitis, multiple proinflammatory cytokines within the intestinal tissue abnormally activate STAT3, resulting in intestinal barrier dysfunction, hyperactivation of innate immunity, and enhanced Th17-mediated immune responses, all of which contribute to chronic intestinal inflammation (Chen X. et al., 2023; Hu et al., 2024). Additionally, SIRT1, a key player in cellular metabolism and stress response, interacts with the STAT3 signaling cascade (Wu Q. J. et al., 2022; Yang et al., 2022; Huang et al., 2024), thereby modulating the inflammatory response in colitis. However, the specific mechanisms underlying this interaction in UC remain incompletely understood. Investigating the SIRT1/STAT3 axis in colitis may provide critical insights for developing novel IBD therapies. Our findings indicated that the protective effect of GHK-Cu against DSS-induced intestinal inflammation depends on the SIRT1/STAT3 signaling pathway (Figure 10).

Notably, activators of SIRT1, such as metformin, have demonstrated the ability to inhibit Th17 cell differentiation and downregulate IL-17A and RORγt levels by promoting STAT3 deacetylation (Hamaidi and Kim, 2022). Furthermore, the differentiation of Th17 cells is significantly influenced by the extent of STAT3 deacetylation (Zou et al., 2020), which ultimately suppresses the induction of RORγt transcription (Limagne et al., 2017). Our study found that GHK-Cu treatment reversed DSS-induced upregulation of RORγt protein, possibly through the SIRT1/STAT3 signaling pathway.

In summary, our study primarily investigated the beneficial effects of GHK-Cu in UC, using a DSS-induced colitis mouse model and an *in vitro* co-culture system, suggesting that GHK-Cu may exert anti-inflammatory properties and its role in facilitating the repair of UC by regulating SIRT1/STAT3 signaling pathway. Our findings could pave the way for a novel preventive strategy against IBD and provide a safe and efficacious therapeutic approach for UC. However, this study has certain limitations that warrant further investigation. The DSS-induced UC mouse model is a short-term model. In the next step, we will construct a chronic enteritis model to further study the GHK-Cu’s effects on chronic enteritis. The study only employed a single drug dose, overlooking dose-dependent investigations, which will also be addressed in subsequent research. Additionally, the exploration of whether the drug affects Th17 cell activity by influencing STAT3 activity remains insufficiently thorough. Further studies will employ multiple indicators for a more comprehensive analysis. Additionally, using SIRT1 knockout mice and STAT3 knockout mouse models may provide more conclusive insights into GHK’s mechanism of action.

## Data Availability

The raw data supporting the conclusions of this article will be made available by the authors, without undue reservation.
