# Supplementary material for: Exploring the beneficial effects of GHK-Cu on an experimental model of colitis and the underlying mechanisms
Source: Front Pharmacol. 2025 Jul 2;16:1551843. doi: 10.3389/fphar.2025.1551843 (PMC12263609; doi:10.3389/fphar.2025.1551843)
Supplement: Supplementary file 1 [file Supplementaryfile1.docx]

Supplementary Material

## Supplementary Figure 1


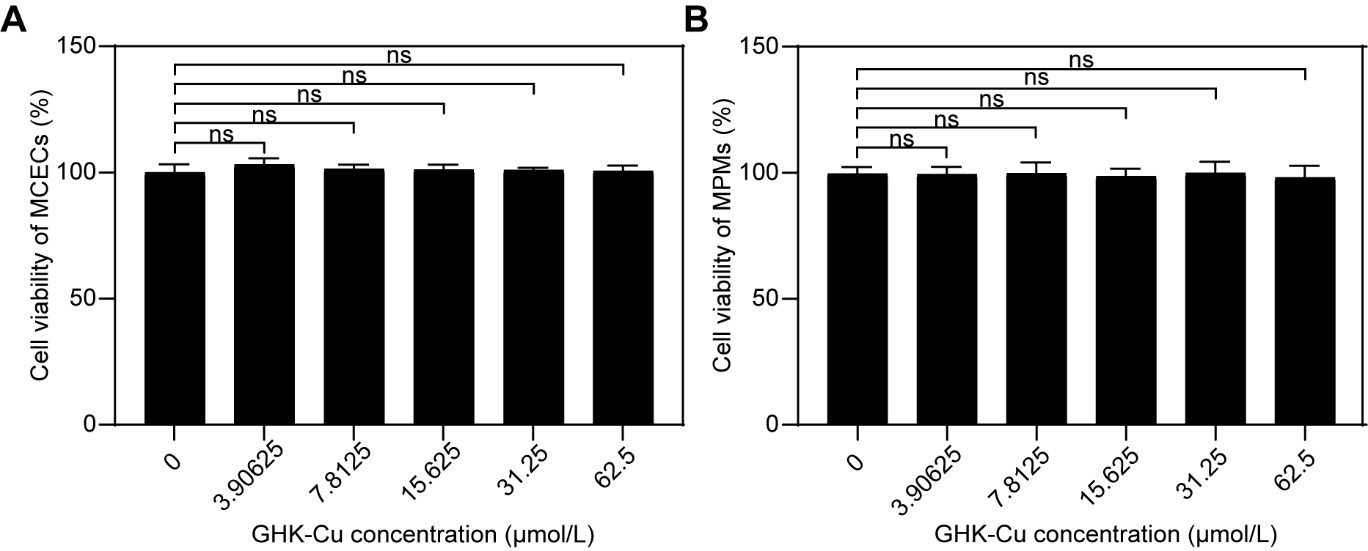


**Supplementary Figure 1.** **The effect of GHK-Cu on the cell viability of MCECs and MPMs as assessed by the MTT assay.** (A) Cell viability of mouse colonic epithelial cells (MCECs) after treatment with varying concentrations of GHK-Cu for 24 hours. (B) Cell viability of mouse peritoneal macrophages (MPMs) after treatment with varying concentrations of GHK-Cu for 24 hours. Results are expressed as a percentage of the untreated control group (0 μmol/L). Data are presented as the mean ± SD (n = 6). Statistical analysis was performed using one-way ANOVA followed by Dunnett's test. "ns" indicates no significant difference between groups (*P* > 0.05).
