# Supplementary figures and images for: Exploring the beneficial effects of GHK-Cu on an experimental model of colitis and the underlying mechanisms
Source: Front Pharmacol. 2025 Jul 2;16:1551843. doi: 10.3389/fphar.2025.1551843 (PMC12263609; doi:10.3389/fphar.2025.1551843)

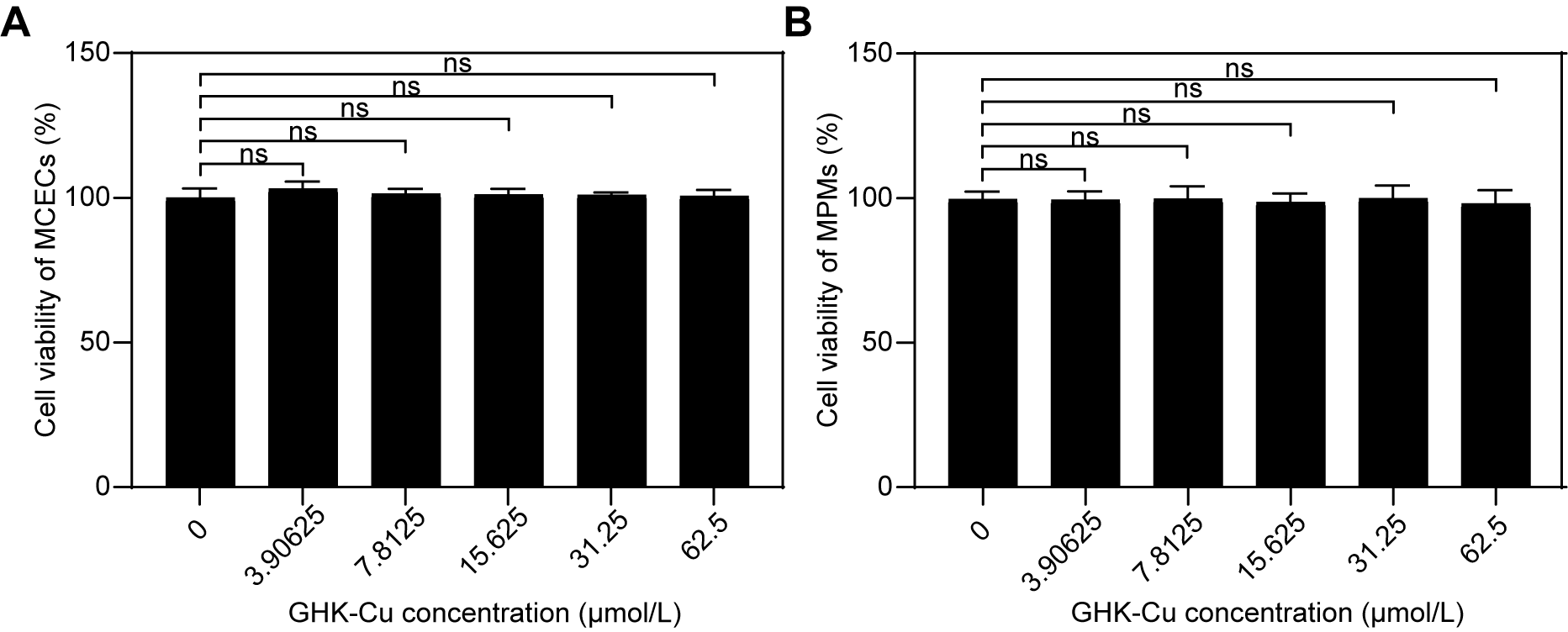

Supplement: Supplementary file 2 [file Image1.tif]
